# Predictive Value of 5-Methoxytryptophan on Long-Term Clinical Outcome after PCI in Patients with Acute Myocardial Infarction-a Prospective Cohort Study

**DOI:** 10.1007/s12265-024-10518-6

**Published:** 2024-04-29

**Authors:** Kui Huang, Xiao-Qin Wen, Wei Zhang, Jing-Xian Wang, Yan Liang, Wen-Qing Li, Yu-Hang Wang, Miao-Miao Liang, An-Ran Jing, Jing Ma, Xu Zhang, Yin Liu, Jing Gao

**Affiliations:** 1 Thoracic Clinical College, Tianjin Medical University, No.22 Qi Xiangtai Road, Heping District, Tianjin, 300070 People’s Republic of China; 2 Department of Cardiology, Tianjin Chest Hospital, No.261 Tai Erzhuang Road, Jinnan District, Tianjin, 300222 People’s Republic of China; 3 Department of Cardiology, Tianjin Hospital, Tianjin, People’s Republic of China; 4 Cardiovascular Institute, Tianjin Chest Hospital, No.261 Tai Erzhuang Road, Jinnan District, Tianjin, 300222 People’s Republic of China; 5 Tianjin Key Laboratory of Cardiovascular Emergency and Critical Care, Tianjin Municipal Science and Technology Bureau, Tianjin, People’s Republic of China; 6 Chest Hospital, Tianjin University, No.92 Weijin Road Nankai District, Tianjin, 300072 People’s Republic of China

**Keywords:** Acute myocardial infarction, Heart failure, HFpEF, MACE, 5-Methoxytryptophan

## Abstract

**Background:**

In recent years, 5-Methoxytryptophan (5-MTP) has been identified as an endothelial factor with vaso-protective and anti-inflammatory properties.

**Methods:**

In this prospective cohort study, a total of 407 patients with acute myocardial infarction (AMI) who underwent percutaneous coronary intervention (PCI) successfully were enrolled. A 1-year follow-up Kaplan–Meier survival analysis was used for evaluating the correlation between 5-MTP and major adverse cardiovascular event (MACE) while Cox proportional-hazards regression was used to identify predictive values of 5-MTP on MACE after AMI.

**Results:**

Increased 5-MTP level led to a significant downtrend in the incidence of MACE (All Log-rank *p* < 0.05). Thus, a high baseline 5-MTP could reduce the 1-year incidence of MACE (HR = 0.33, 95%Cl 0.17–0.64, *p* = 0.001) and heart failure (HF) (HR = 0.28, 95% Cl 0.13–0.62, *p* = 0.002). Subgroup analysis indicated the predictive value of 5-MTP was more significant in patients aged ≤ 65 years and those with higher baseline NT-proBNP, T2DM, STEMI, and baseline HF with preserved LVEF (HFpEF) characteristics.

**Conclusions:**

Plasma 5-MTP is an independent and protective early biomarker for 1-year MACE and HF events in patients with AMI, especially in younger patients and those with T2DM, STEMI, and baseline HFpEF characteristics.

**Graphical Abstract:**

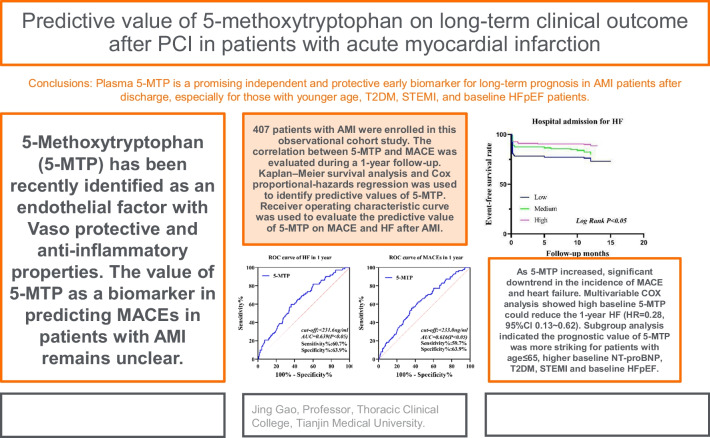

**Supplementary Information:**

The online version contains supplementary material available at 10.1007/s12265-024-10518-6.

## Introduction

Cardiac disease is the leading cause of death in both urban and rural residents in China. High long-term mortality is mostly caused by HF and other MACE following AMI [1]. PCI and guideline-directed medical therapy can reduce the in-hospital mortality of AMI, however, ischemic heart disease increases the prevalence of HF [1]. Therefore, early identification of such high-risk patients is crucial. Age, body weight, and renal function all have an impact on traditional HF biomarkers, such as Brain Natriuretic Peptide (BNP) or N-terminal pro-BNP (NT-proBNP) [2, 3]. Therefore, the exploration of new biomarkers that can early predict HF after AMI has garnered great attention in recent years.

5-MTP, an endogenous tryptophan metabolite, is synthesized from L-tryptophan via two enzymatic steps: tryptophan hydroxylase-1 (TPH-1) catalyzes the initial step which converts L-tryptophan to 5-hydroxytryptophan (5-HTP) and hydroxy indole O-methyltransferase (HIOMT) catalyzes the last step which converts 5-HTP to 5-MTP. 5-MTP is synthesized in human fibroblasts, vascular endothelial cells, vascular smooth muscle cells, bronchial epithelial cells as well as renal epithelial cells, and secreted into the blood via endoplasmic reticulum’s Golgi system to exert physiological effects [4]. It can also inhibit the transcriptional expression of cyclooxygenase-2 (COX-2); thus, inhibiting inflammation, proliferation, migration, and invasion of tumor cells [5, 6]. Simultaneously, 5-MTP can inhibit the bacterial lipopolysaccharide-induced systemic inflammatory response and alleviate tissue injury as well as fibrosis by inhibiting COX-2, monocyte chemoattractant protein-1 (MCP-1), interleukin-6 (IL-6) and nuclear factor kB (NF-kB) expressions [5, 7].

Additionally, 5-MTP can inhibit the proliferation and migration of tumor cells by hampering the p38-MAPK/NF-kB pathway, stenosis caused by the vascular injury-induced proliferation of endothelial and smooth muscle cells, chronic kidney disease (CKD) progression as well as renal and liver fibrosis as seen in various studies on tumorigenesis [8], inflammation inhibition [9], peripheral artery disease [10], CKD [11], and liver fibrosis [12].

A cardiovascular study showed lower myocardial TPH-1 mRNA and plasma 5-MTP levels 24 and 48 h after coronary artery ligation, suggesting that the protective effects of 5-MTP were attenuated post-myocardial infarction (MI). Subsequently, an intraperitoneal 5-MTP injection can reduce post-MI myocardial injury, infarct size, and fibrosis as well as restore myocardial function [13]. Presently, the only clinical study [14] to evaluate the predictive value of 5-MTP in post-MI left ventricular remodeling (LVR) found that plasma 5-MTP levels were lower in ST-segment elevation myocardial infarction (STEMI) patients and 5-MTP level at day 3 post-MI significantly correlated with the NT-proBNP level 1-year post-MI, suggesting the efficacy of 5-MTP in predicting the incidence of HF after MI, however, no clinical adverse event was included in the end point of observation.The association between 5-MTP levels and long-term clinical clinical outcomes after PCI in patients with AMI remains unknown.Thus, our study aimed to observe the predictive value of 5-MTP in long-term clinical MACE and HF characteristics after PCI in patients with AMI.

## Materials and Methods

### Study Population

We included all patients diagnosed with AMI by the cardiac care unit (CCU) in Tianjin Chest Hospital and successfully treated with PCI from October 2018 to October 2019.

The diagnosis of AMI was based on the fourth universal definition of MI criteria [15]:

1. STEMI: a) elevated serum cardiac necrosis markers (mainly troponin) (at least exceeding the upper limit of 99% reference value); b) New ST-segment elevation at the J-point in ≥ 2 contiguous leads on the electrocardiogram (ECG); and c) One or more of the following features: persistent ischemic chest pain and imaging displaying new regional wall motion abnormality as well as abnormal coronary angiography.
2. non-ST elevation myocardial infarction (NSTEMI): a) Cardiac troponin > upper limit of 99% reference value, and b) ECG showing an ST-segment depression or T-wave inversion, and/or persistent chest pain for > 30 min.

**Fig. 1:**
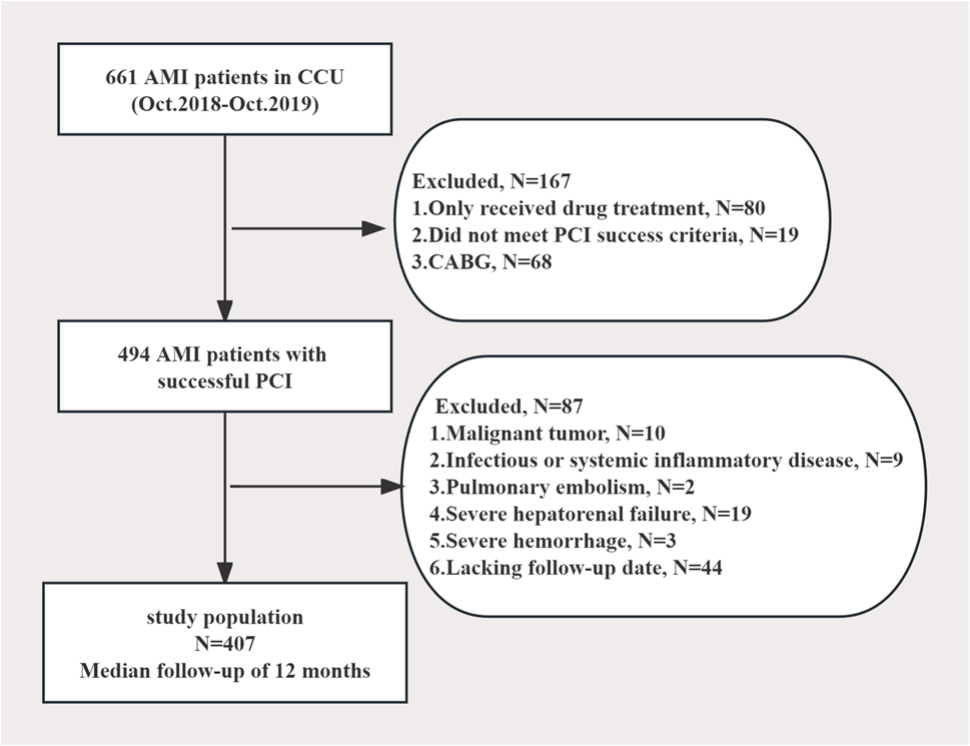
Flowchart showing the selection process and excluded patients. AMI, acute myocardial infarction; CABG, coronary artery bypass graft; PCI, percutaneous coronary intervention

The exclusion criteria were: (1) Patients who did not undergo PCI or did not meet PCI success criteria; (2) Those with malignant tumors, infectious or systemic inflammatory diseases; (3) Patients with chest pain caused by other heart diseases or non-cardiac causes like myocarditis, aortic dissection, pulmonary embolism, etc.; (4) Those with other complications like severe hepatorenal failure, hemorrhage, thrombocytopenia and stroke within one month; (5) Patients with cognitive impairment and inability to communicate with experimental observation or refusal to sign informed consent form, and (6) Pregnant women. A total of 407 patients were included who met the inclusion and exclusion criteria; Fig. 1 depicts the study’s flowchart.

Ethics: Informed consent was obtained from each patient. The study protocol conformed to the ethical guidelines of the 1975 Declaration of Helsinki and was approved by the Ethics Committee of Tianjin Chest Hospital (No. 2018KY-010–01).

### Clinical Data Collection and PCI Treatment

Sex, age, body mass index (BMI), previous medical history, emergency laboratory indexes of admission, like creatine kinase (CK), creatine kinase MB (CK-MB), high-sensitivity troponin T (hsTnT), NT-proBNP, serum creatinine (Cr), heart rate (HR), sitting blood pressure (measured on the non-dominant arm supported at the heart level), and Killip classification were collected on initial presentation to CCU by senior doctors blinded to the study’s purpose. The triglyceride (TG), high-density lipoprotein cholesterol (HDL-C), low-density lipoprotein cholesterol (LDL-C), and total cholesterol (TC) were measured after a 12-h fast. EpiData software was used for double data entry with adequate checks followed by validation.

BMI was defined as weight (kg)/[height (m)^2^]. Hypertension was characterized based on repeated office systolic blood pressure (SBP) values ≥ 140 mmHg and/or diastolic blood pressure (DBP) ≥ 90 mmHg [16]. According to ADA 2019 [17] diabetes was defined as symptoms with recurring fasting plasma glucose (FPG) ≥ 7.0 mmol/L (126 mg/dL) or 2 h plasma glucose (2 h PG) ≥ 11.1 mmol/L (≥ 200 mg/dL) or symptoms plus random plasma glucose (RPG) ≥ 11.1 mmol/L (≥ 200 mg/dL). Individuals who smoked or had a previous smoking history with one cigarette per day for at least six months were considered smokers.

Blood sample collection: Median cubital vein blood samples were collected early in the morning after a 12-h fast on admission. After storing blood samples temporarily at 4 °C, plasma (2 mL) was extracted within 2 h by centrifugation under 3000 rpm, and frozen at –80 °C in Tianjin Chest Hospital Biobank until detection. Moreover, plasma 5-MTP levels were detected by ELISA using a commercial kit (R&D Systems, Minneapolis, United States) [14].

PCI was performed by two cardiologists blinded to the study’s purpose according to international standards and guidelines [18], disagreement was resolved by consensus. An emergency PCI was advised for STEMI patients to satisfy the door to balloon (D to B) time < 90 min. According to the risk stratification [18],  NSTEMI patients underwent an immediate invasive approach (PCI < 2 h after admission), early PCI (2–24 h after admission), delayed PCI (24–72 h after admission), or late PCI (≥ 72 h post-admission). Only culprit lesion PCI was performed on patients with non-cardiogenic shock. Unfractionated heparin (70 to 100 U/kg) was given before or during the PCI to maintain the activated clotting time (ACT) at 250 to 300 s. After PCI, a Swan-Ganz catheter was inserted into the jugular vein to monitor hemodynamics. A maintenance dose of ticagrelor (90 mg, twice daily) or clopidogrel (75 mg, once daily) and aspirin (100 mg, once daily) was prescribed.

PCI success criteria included significant lumen enlargement at the stenosis site after stenting, residual lumen diameter < 20%, the Thrombolysis in Myocardial Infarction (TIMI) grade 3 blood flow, and the absence of crucial complications such as death, MI, and emergency target lesion revascularization (TLR) during hospitalization.

Left atrial diameter (LAD), left ventricular end-diastolic diameter (LVED), left ventricular ejection fraction (LVEF), etc. were evaluated by analysts blinded to study’s purpose within 48 h after AMI as per the clinical standards and current echocardiographic guidelines  [19].

All imaging equipment used for PCI and echocardiography was calibrated regularly. Frequent quality checks as well as validation measurements were done to strengthen the study’s robustness.

### Follow-Up and Observation End-Points

Prospective follow-up was carried out by clinic visits or telephone interviews at 1, 3, 6, and 12 months from the date of selection. The staff regularly followed up with the AMI patients and urged them for timely visits.

The primary end-points were MACE within 1 year after discharge, including all-cause death, recurrent non-fatal MI, TLR, HF hospitalization, and stroke. TLR was defined as either PCI or coronary artery bypass grafting (CABG) resulting from restenosis or thrombosis of the stent-treated target lesion that included the proximal and distal edge segments and the side branch ostium. HF during follow-up was defined as admission due to suspected symptoms of HF with (1). Suspected symptoms and signs of HF; (2). Chest X-ray showed pulmonary congestion, pulmonary edema, or cardiac enlargement; (3). NT-proBNP ≥ 300 pg/mL, and (4). Excluding symptoms of other causes [19]. Stroke during a follow-up was defined as ischemic or hemorrhagic stroke requiring hospitalization with symptoms lasting 24 h.

All end-points were adjudicated centrally by 2 independent cardiologists, and disagreement was resolved by consensus. Additionally, regular quality checks, repeated measurements, and internal laboratory quality control measures were carried out to ensure research quality during the process of data collection and analysis.

## Statistical Analysis

All calculations were performed with SPSS 25.0 software (IBM, Munich, Germany). Since all variables were not normally distributed after the Shapiro–Wilk test, median (Q1,Q3), non-parametric Kruskal–Wallis, or Mann-Whiney U tests were used for comparison between groups. The categorical variables were expressed as n (%), and differences between groups were compared using Pearson’s Chi-square or Fisher’s exact tests. The Bonferroni method was used for multiple comparisons while Spearman’s rank correlation analysis was used to examine the correlation between 5-MTP, other biomarkers, and detection indexes.

For follow-up events, Cox regression analysis analyzed the effects of different 5-MTP levels and related risk factors on long-term MACE events in patients with AMI while the Kaplan–Meier method was used to build the survival curve, and a log-rank test was used to evaluate its statistical difference. The receiver operating characteristic curve (ROC) evaluated the predictive value of 5-MTP concentration on MACE and HF after AMI. The subgroup classifications were: 407 patients by male vs. female; age ≤ 65 years vs. age > 65 years; T2DM vs. non-T2DM; STEMI vs. NSTEMI; High NT-proBNP vs. Low NT-proBNP, and 267 patients with HF during hospitalization by HF with reduced LVEF (HFrEF) vs. HF with mildly reduced LVEF (HFmrEF) vs. HFpEF respectively. Two-sided p-values < 0.05 were considered statistically significant. All figures were constructed using GraphPad Prism version 9.0.0 for Windows (GraphPad Software, San Diego, California USA, www.graphpad.com).

## Results

### Selection of the 5-MTP Cut-Off Point

The 5-MTP concentrations were divided into three groups according to X-tile (Yale University, New Haven, CT, United States) [20]: low (149.8–204.9 ng/mL), medium (204.9–266.5 ng/mL) and high (266.5–367.5 ng/mL) to achieve the homogeneity and the heterogeneity of intra-group as well as inter-group survival rates, respectively.

### Clinical Baseline Data

Table 1 shows the baseline characteristics of 407 patients grouped by 5-MTP concentration. With increased 5-MTP levels, NT-proBNP showed a downward trend, LVEF showed an upward trend, and the proportion of Killip I increased, while the proportion of Killip II ~ III and IV grades decreased. Significant differences were observed in NT-proBNP, LVEF, and Killip classifications among the three groups (*p* < 0.05).

**Table 1:** Patients’ baseline and follow-up data grouped according to 5-MTP levels

	All patients (*n* = 407)	5-MTP < 204.9 (Low) (*n* = 106)	204.9 ≤ 5-MTP ≤ 266.5 (Medium) (*n* = 120)	5-MTP > 266.5 (High) (*n* = 181)	***p***
Ages (years)	60.0(45.0,69.0)	58.5(44.8,69.0)	59.0(45.0,69.0)	60(45.0,69.0)	0.878
Male, n(%)	291(71.5)	70(66.0)	92(76.7)	129(71.3)	0.209
BMI(kg/m^2^)	25.57(23.89,26.84)	25.62(24.67,26.97)	25.82(23.87,27.51)	25.39(23.67,26.57)	0.157
≥ 28 kg/m^2^,*n* (%)	49(15.4)	15(17.4)	17(18.1)	17(12.2)	0.382
SBP(mmHg)	131.0(120.0,146.0)	128.5(115.0,150.0)	130.0(120.0,143.25)	135.0(120.0,147.5)	0.199
HR(bpm)	73(65,82)	77(66,90)	71(65,81)	70(65,80)	0.004
AMI types, *n* (%)					0.820
STEMI	290(71.3)	78(73.6)	85(70.8)	127(70.2)	
NSTEMI	117(28.7)	28(26.4)	35(29.2)	54(29.8)	
History, *n* (%)					
Smoker	239(58.7)	69(65.1)	67(55.8)	103(56.9)	0.296
Hypertension	241(59.2)	60(56.6)	78(65.0)	103(56.9)	0.307
Diabetes	117(28.7)	40(37.7)	30(25.0)	47(26.0)	0.058
Hyperlipidemia	150(36.9)	45(42.5)	42(35.0)	63(35.0)	0.393
MI	51(12.5)	15(14.2)	16(13.3)	20(11.0)	0.709
PCI	49(12.0)	13(12.3)	13(10.8)	23(12.7)	0.884
CABG	20(4.9)	7(6.6)	6(5.0)	7(3.9)	0.585
Biochemical characteristics, median (Q1, Q3)					
WBC, × 10^9^/L	9.53(7.46,12.41)	9.67(6.99,12.65)	9.52(7.68,12.08)	9.44(7.35,12.36)	0.924
PLT, × 10^9^/L	221.5(184.0,269.0)	224.5(184.3,265.0)	219.5(186.3,277.3)	222.0(181.5,266.8)	0.919
CK(U/L)	709.00 (149.25,1863.75)	650.50 (145.50,2095.00)	715.50 (207.00,1909.25)	676.50 (116.25,1796.25)	0.369
CK-MB(U/L)	63.00 (22.00,168.25)	59.00 (22.75,174.75)	70.00 (23.25,187.00)	61.50 (20.25,167.25)	0.668
hsTNT(ng/mL)	1.45(0.32,4.74)	1.34(0.36,6.44)	1.50(0.35,4.80)	1.54(0.25,4.47)	0.903
NT-proBNP (pg/mL)	773.60 (244.26,1537.00)	975.30 (368.80,1866.00)	764.40 (208.80,1802.00)	670.40 (194.45,1417.50)	0.029
hsCRP(mg/L)	5.14(2.02,15.64)	5.34(2.07,15.45)	5.16(1.90,13.64)	4.94(2.02,21.01)	0.985
> 5 mg/L, n(%)	209(51.6)	95(52.8)	62(51.7)	52(49.5)	0.866
TG (mmol/L)	1.58(1.15,2.25)	1.65(1.19,2.39)	1.57(1.132.35)	1.52(1.15,2.14)	0.676
TC (mmol/L)	4.45(3.75,5.22)	4.43(3.77,5.26)	4.56(3.84,5.26)	4.45(3.65,5.13)	0.353
LDL (mmol/L)	2.91(2.27,3.64)	2.90(2.33,3.74)	2.90(2.33,3.65)	2.92(2.20,3.48)	0.592
HDL (mmol/L)	1.00(0.84,1.21)	0.96(0.81,1.19)	1.02(0.85,1.22)	1.01(0.85,1.21)	0.418
UA (mmol/L)	322.00 (263.00,379.50)	310.00 (249.50,370.00)	339.00 (285.25,392.75)	319.00 (259.00,381.00)	0.057
Cr(µmol/l)	76.00(65.00,89.00)	74.00(64.50,87.00)	77.00(64.00,90.75)	76.00(66.50,88.00)	0.615
Killip classification on admission, *n* (%)					0.042
I	366(90.1)	92(86.8)	105(87.5)	169(93.9)	
II ~ III	38(9.4)	12(11.3)	15(12.5)	11(6.1)	
IV	2(0.5)	2(1.9)	0(0.0)	0(0.0)	
LVEF (%)	50(44,55)	48(41,50)	50(45,55)	50(45,56)	< 0.001
≤ 40, n (%)	67(16.9)	23(22.3)	21(17.8)	23(13.1)	0.179
HF during hospital admission Based on LVEF (*n* = 267), *n* (%)					0.083
HFrEF	61(22.8)	23(29.5)	19(24.4)	19(17.1)	
HFmrEF	92(34.5)	31(39.7)	24(30.8)	37(33.3)	
HFpEF	114(42.7)	24(30.8)	35(44.9)	55(49.5)	
Medication, *n* (%)					
DAPT	406(99.8)	106(100)	120(100)	180(99.4)	0.535
Statin	403(99.0)	105(99.1)	120(100)	178(98.3)	0.361
ACEI/ARB	352(86.5)	89(84.0)	109(90.8)	154(85.1)	0.244
Beta-blocker	368(90.4)	94(88.7)	111(92.5)	163(90.1)	0.607
Coronary lesion, *n* (%)					
Single lesion	248(60.9)	71(71.7)	65(61.3)	112(69.1)	0.241
Double lesions	47(11.5)	10(10.1)	16(15.1)	21(13.0)	0.563
Three lesions	65(16.0)	17(17.2)	23(21.7)	25(15.4)	0.416
Left main	29(7.1)	5(5.0)	9(8.1)	15(8.7)	0.518
Outcome in 1 year, *n* (%)					
MACEs	88(21.6)	32(30.2)	29(24.2)	27(14.9)	0.007
HF hospitalization	72(17.7)	28(26.4)	24(20.0)	20(11.0)	0.003
All-cause death	10(2.5)	4(3.8)	3(2.5)	3(1.7)	0.555
Non-fatal recurrent MI	7(1.7)	3(2.8)	2(1.7)	2(1.1)	0.556
TLR	6(1.5)	2(1.9)	2(1.7)	2(1.1)	0.879
stroke	2(0.5)	1(0.9)	1(0.8)	0(0.0)	0.308

*ACEI* angiotensin-converting enzyme inhibitor, *ARB* angiotensin receptor blocker, *BMI* body mass index, *CABG* coronary artery bypass graft, *Cr* creatinine, *CK* creatine kinase, *CK-MB* creatine kinase MB, *DAPT* dual antiplatelet therapy, *HDL-C* high-density lipoprotein cholesterol, *HF* heart failure, *HFrEF* HF with reduced EF(LVEF ≤ 40%), *HFmrEF* HF with mildly reduced EF(LVEF:41–49), *HFpEF* HF with preserved EF(LVEF ≥ 50%), *HR* heart rate, *hsCRP* high sensitivity C-reactive protein, *hsTnT* high-sensitivity troponin T, *LDL-C* low-density lipoprotein cholesterol, *LVEF* left ventricular ejection fraction, *MACE *major adverse cardiovascular event, *5-MTP* 5-methoxytryptophan, *MI* myocardial infarction, *NSTEMI* non-ST elevated myocardial infarction, *NT-proBNP* N-Terminal pro-brain natriuretic peptide, *PCI* percutaneous coronary intervention, *PLT* platelet count, *SBP* systolic blood pressure, *STEMI* ST elevated myocardial infarction, *TC* total cholesterol, *TG* triglyceride, *TLR* target lesion revascularization, *WBC *white blood cell, *UA* Uric Acid

**Fig. 2:**
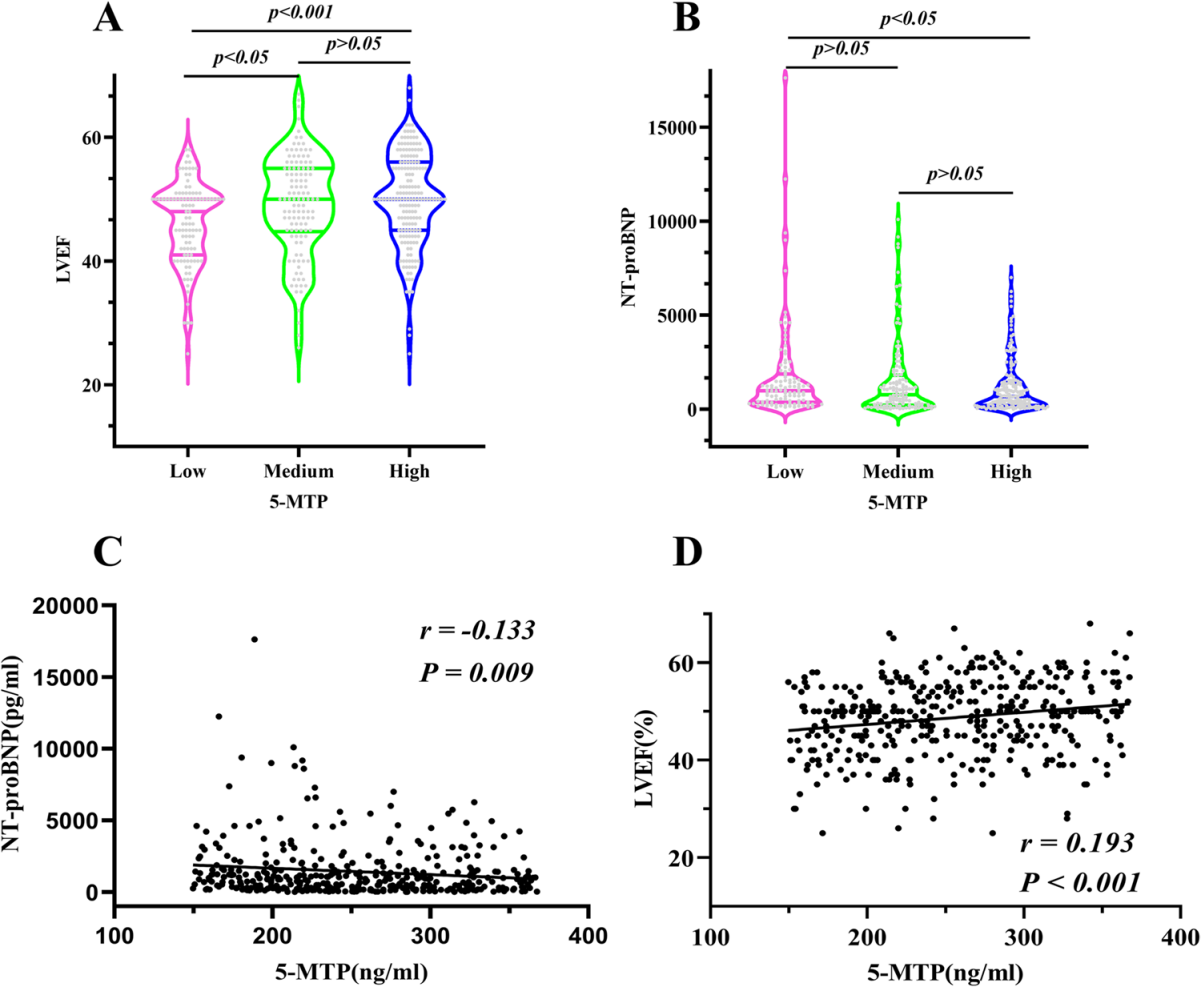
(**A**, **B**) Multiple comparisons of NT-proBNP and LVEF among three groups with high, middle, and low 5-MTP concentrations. (**C**, **D**) Correlation between 5-MTP and NT-proBNP and 5-MTP as well as LVEF, respectively. LVEF, left ventricular ejection fraction; 5-MTP, 5-methoxytryptophan; NT-proBNP, N-Terminal pro-brain natriuretic peptide

Multiple comparisons showed statistical differences in NT-proBNP between the high and low 5-MTP groups, respectively. Although difference of LVEF between low and middle as well as low and high groups was statistically significant, no significant difference was noticed in LVEF between the middle and high groups, respectively (Fig. 2A, B).

HF during hospitalization or baseline HF (*n* = 267) was characterized by the symptoms or signs of suspected HF during hospitalization and NT-proBNP ≥ 300 pg/mL, excluding other causes. According to LVEF, it was divided into HFrEF (LVEF ≤ 40%), HFmrEF (LVEF:41–49), and HFpEF (LVEF ≥ 50%) [19]. The diagnosis of HFpEF also fulfilled the echocardiographic determination criteria with E/e' ≥ 15 or resting cardiac catheterization indicating pulmonary capillary wedge pressure (PCWP) ≥ 15 mmHg.

### Correlation between 5-MTP, other Biomarkers, and LVEF

Spearman’s rank correlation analysis showed no significant correlation between 5-MTP and CK, CK-MB, hsTNT, hsCRP, WBC, and other biomarkers, respectively. However, 5-MTP was significantly correlated with NT-proBNP (*r* = –0.133, *p* < 0.05) and LVEF (*r* = 0.193,* p* < 0.001, Fig. 2 C, D).

### Group Comparison based on MACE

**Fig. 3:**
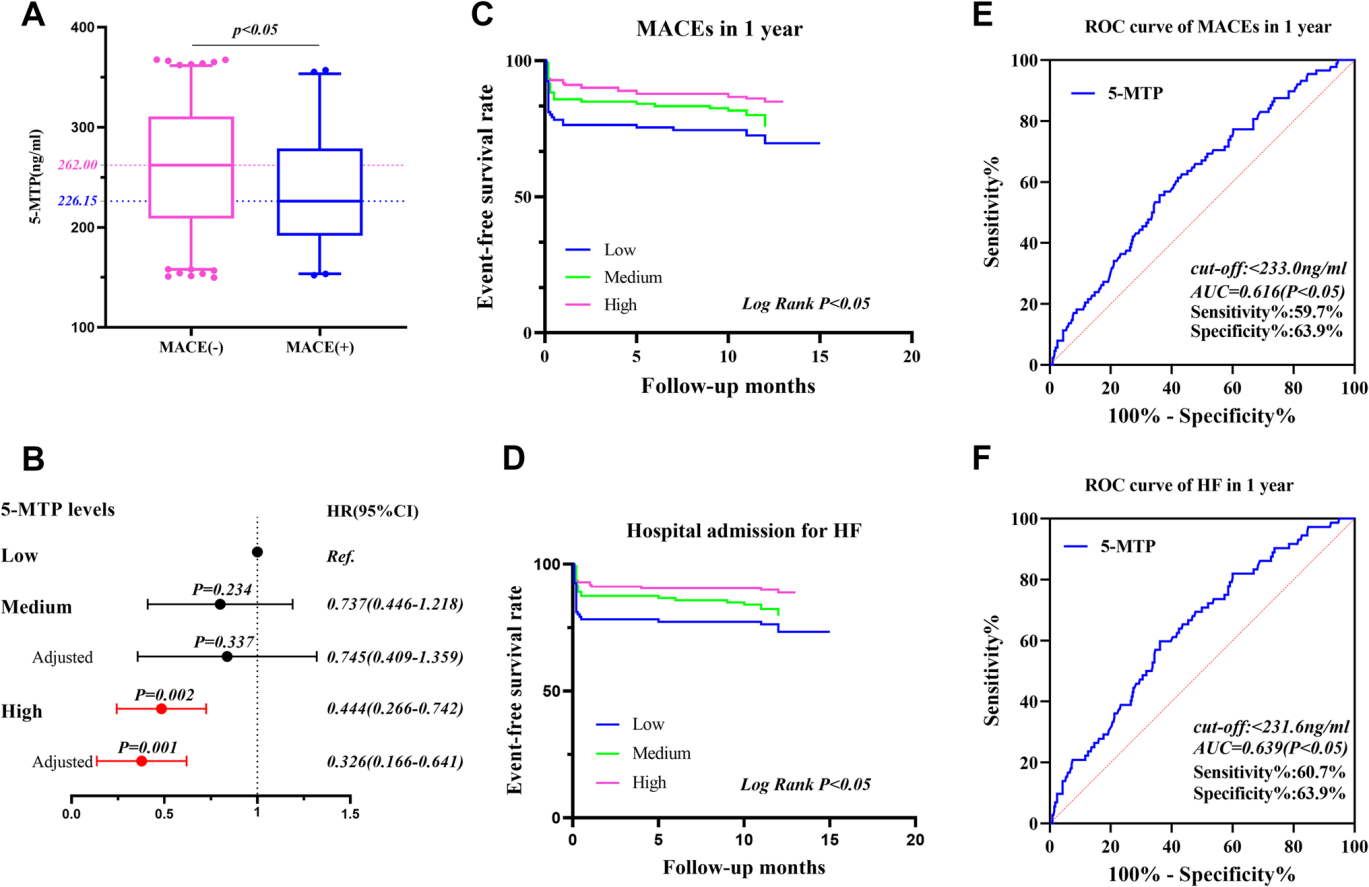
(**A**) Comparison of 5-MTP levels in each group based on MACEs. (**B**) Forest plot shows univariate and multivariable COX regression analyses and the HRs with 95% CIs for MACE adjusted for sex, age, hypertension, diabetes, MI history, NT-proBNP, Cr, hsCRP, LVEF, triple vessel disease and left main artery disease. (**C**, **D**) Kaplan–Meier survival curves of MACEs and HF in 407 patients with AMI. (**E**, **F**) ROC curve of baseline 5-MTP concentration to predict 1-year MACE and HF of patients with AMI after discharge

Table 2 shows that the MACE ( +) group was older with a higher proportion of diabetes, MI, three-vessel disease, and left main lesion. Additionally, the MACE ( +) group’s NT-proBNP (pg/mL) [2070.5 (1106.75, 4649.25) vs. 491.8 (165.3, 1201.0), *p* < 0.05], Cr (µmol/l) [82 (65,108) vs. 73 (65,86),* p* < 0.05], and HsCRP (mg/L) [16.29 (5.14,74.95) vs. 5.52 (2.53, 13.24), *p* < 0.05] was significantly higher than the MACE (-) group. The MACE ( +) patients’ LVEF was reduced [45 (40,51) vs. 50 (46.5,56), *p* < 0.001]. Moreover, the 5-MTP level was significantly reduced in MACE ( +) patients than in MACE (-) patients [226.15 (191.53,278.80) ng/mL vs. 262.00 (208.80,310.80) ng/mL, *p* < 0.05, Fig. 3A].

**Table 2:** Patients’ baseline characteristics stratified by MACEs

Characteristics	MACE(-)(n = 319)	MACE(+) (*n* = 88)	*x*^2^/Z	*P*
Male, *n* (%)	235(73.7)	56(63.6)	3.41	0.082
Ages (years)	57.00(45.00,67.00)	67.00(52.00,76.00)	-2.96	0.003
BMI (kg/m^2^)	25.58(23.87,26.84)	25.54(24.09,27.04)	-0.31	0.760
History, n (%)				
Hypertension	183(57.4)	58(65.9)	2.08	0.149
Diabetes	75(23.5)	42(47.7)	19.75	< 0.001
Smoking	192(60.2)	47(53.4)	1.31	0.253
Hyperlipidemia	122(38.2)	28(32.2)	1.08	0.299
MI	30(9.4)	21(23.9)	13.16	< 0.001
Biochemical characteristics, median (Q1, Q3)				
WBC, × 10^9^/L	9.53(7.48,12.16)	9.39(7.19,12.53)	-0.02	0.983
Hb(g/L)	140.00(128.00,151.00)	138.00(125.00,154.00)	-4.42	0.672
PLT, × 10^9^/L	223.00(188.00,269.00)	203.00(169.00,266.00)	-1.01	0.315
hsTNT (ng/mL)	1.46(0.35,4.51)	1.39(0.28,5.38)	-0.109	0.913
NT-proBNP,(pg/mL)	491.80(165.30,1201.00)	2070.50(1106.75,4649.25)	-8.15	< 0.001
TG (mmol/L)	1.93(1.35,2.70)	1.98(1.27,2.42)	-0.86	0.389
TC (mmol/L)	4.70(3.93,5.27)	4.60(3.80,5.31)	-0.46	0.644
LDL (mmol/L)	2.91(2.30,3.69)	3.21(2.60,3.64)	-1.40	0.161
Cr (µmol/l)	73.00(65.00,86.00)	82.00(65.00,108.00)	-2.69	0.007
HsCRP (mg/L)	5.52(2.53,13.24)	16.29(5.14,74.95)	-3.36	0.001
Transthoracic echocardiography, median (Q1, Q3)				
LAD(mm)	37(34,39)	38(35,41)	-1.67	0.096
LVEDD(mm)	52(49,55)	53(49,56)	-1.60	0.110
LVEF(%)	50(47,56)	45(40,51)	-6.12	< 0.001
Medication, *n* (%)				
DAPT	318(99.7)	88(100)	0.28	0.599
Anticoagulant	319(100.0)	86(98.9)	3.68	0.214
Statin	316(99.1)	87(98.9)	0.03	0.869
ACEI/ARB	276(86.5)	73(83.0)	0.72	0.491
Beta-blocker	289(90.6)	77(87.5)	0.73	0.424
Coronary lesion, *n* (%)				
Double-vessel disease	35(11.8)	11(15.5)	0.72	0.425
Triple-vessel disease	49(16.5)	22(31.0)	7.72	0.007
Left main disease	14(4.6)	15(19.2)	19.02	< 0.001
5-MTP(ng/mL)	262.00(208.80,310.80)	226.15(191.53,278.80)	-3.32	0.001
5-MTP level as categorical variables, *n* (%)			9.85	0.007
Low	74(23.2)	32(36.4)		
Medium	91(28.5)	29(33.0)		
High	154(48.3)	27(30.7)		

*ACEI* angiotensin-converting enzyme inhibitor, *ARB* angiotensin receptor blocker, *BMI* body mass index, *DAPT* dual antiplatelet therapy, *HR* heart rate, *hsCRP* high-sensitivity C-reactive protein, *hsTnT* high-sensitivity troponin T, *LAD* left atrial diameter, *LDL-C* low-density lipoprotein cholesterol, *LVEDD *left ventricular end-diastolic dimension, *LVEF* left ventricular ejection fraction, *5-MTP* 5-methoxytryptophan, *MI* myocardial infarction, *NT-proBNP* N-Terminal pro-brain natriuretic peptide, *SBP *systolic blood pressure, *TC* total cholesterol, *TG* triglyceride

### Follow-Up Results

With a median follow-up length of 12 months, all patients were followed up to 1 year after discharge. A total of 88 patients (21.6%) experienced at least one MACE. The incidence of MACE in low, middle, and high 5-MTP groups were 32 (30.2%), 29 (24.2%), and 27 (14.9%), respectively (χ2 = 9.85, *p* < 0.05). Multiple comparisons showed there was a significant difference between the low and high groups, respectively. The incidence rate of HF hospitalization in low, middle, and high 5-MTP groups were 28 (26.4%), 24 (20.0%), and 20 (11.0%), respectively (*p* < 0.05). Multiple comparisons showed that there was a significant difference between the low and high 5-MTP groups, respectively. Additionally, all-cause death occurred in 4 (4.8%), 3 (2.5%), and 3 (1.7%) cases in low, middle, and high groups respectively which was non-significant (*p* > 0.05). Similar results were obtained for other events such as recurrent non-fatal MI, TLR, and stroke (*p* > 0.05, Table 1).

Kaplan–Meier survival curve showed that 5-MTP level was associated with 1-year MACE and HF hospitalization after AMI. Furthermore, the hospital admission for HF event-free survival rate and the 1-year MACE event-free survival rate steadily increased as the 5-MTP levels rose; the difference was statistically significant (all Log Rank *p* < 0.05, Fig. 3C, D).

ROC curve analysis revealed that plasma 5-MTP had a good area under the curve (AUC) to predict MACE (AUC:0.616, 95% Cl 0.551–0.680) and hospital admission for HF (AUC: 0.639, 95% Cl 0.571–0.707) 1-year post-MI. (Fig. 3E, F).

### Predictive Value of Plasma 5-MTP on MACE in One Year after AMI by COX Regression Analysis

The 5-MTP concentration was included in the construction of the univariate COX regression equation. The results showed that in comparison to the low 5-MTP level, the effect of high 5-MTP on 1-year MACE was statistically significant (HR = 0.444, 95% Cl 0.266–0.742, *p* < 0.05, Fig. 3B).

Table 3 shows a multivariable COX regression equation that included traditional risk factors like sex, age, hypertension, diabetes, MI history, NT-proBNP, Cr, hsCRP, LVEF, triple vessel disease, left main artery disease, and 5-MTP. Furthermore, NT-proBNP was adjusted to a binary variable according to 300 pg/mL [19]. The results showed that when compared with low 5-MTP levels, the effect of high 5-MTP level on 1-year MACE was statistically significant (HR = 0.326, 95%Cl 0.166–0.641, *p* = 0.001).

**Table 3:** Multivariable COX regression analysis of the factors influencing MACE 1 year after AMI

Variables	groups	*B*	*SE*	*Wald*	*P*	HR	95%CI
Sex	Female/Male*	0.033	0.316	0.011	0.916	1.034	0.557–1.919
Age		-0.003	0.011	0.064	0.800	0.997	0.976–1.019
Hypertension	Yes/No*	0.431	0.285	2.286	0.131	1.539	0.880–2.692
Diabetes	Yes/No*	1.046	0.290	12.977	< 0.001	2.845	1.611–5.026
Previous MI	Yes/No*	0.115	0.339	0.116	0.734	1.122	0.577–2.182
NT-proBNP	High/Low*	1.511	0.346	19.066	< 0.001	4.533	2.300–8.933
Cr		0.008	0.004	3.627	0.057	1.008	1.000–1.016
hsCRP		0.014	0.006	5.058	0.025	1.015	1.002–1.027
LVEF		-0.088	0.019	22.244	< 0.001	0.916	0.883–0.950
Triple-vessel disease	Yes/No*	0.910	0.327	7.764	0.005	2.484	1.310–4.712
Left main disease	Yes/No*	0.221	0.467	0.224	0.636	1.247	0.449–3.114
5-MTP	Low*						
	Medium	-0.294	0.306	0.920	0.337	0.745	0.409–1.359
	High	-1.121	0.345	10.575	0.001	0.326	0.166–0.641

*Denotes reference group; NT-proBNP ≥ 300 pg/mL was defined as High, otherwise as Low; MI, myocardial infarction; NT-proBNP, N-Terminal pro-brain natriuretic peptide; Cr, creatinine; hsCRP, high-sensitivity C-reactive protein; LVEF, left ventricular ejection fraction

High 5-MTP concentration could reduce the 1-year incidence of HF (HR = 0.279, 95%Cl 0.126–0.619, *p* = 0.002) in comparison with low 5-MTP concentrations.(supplementary Table 1).

**Fig. 4:**
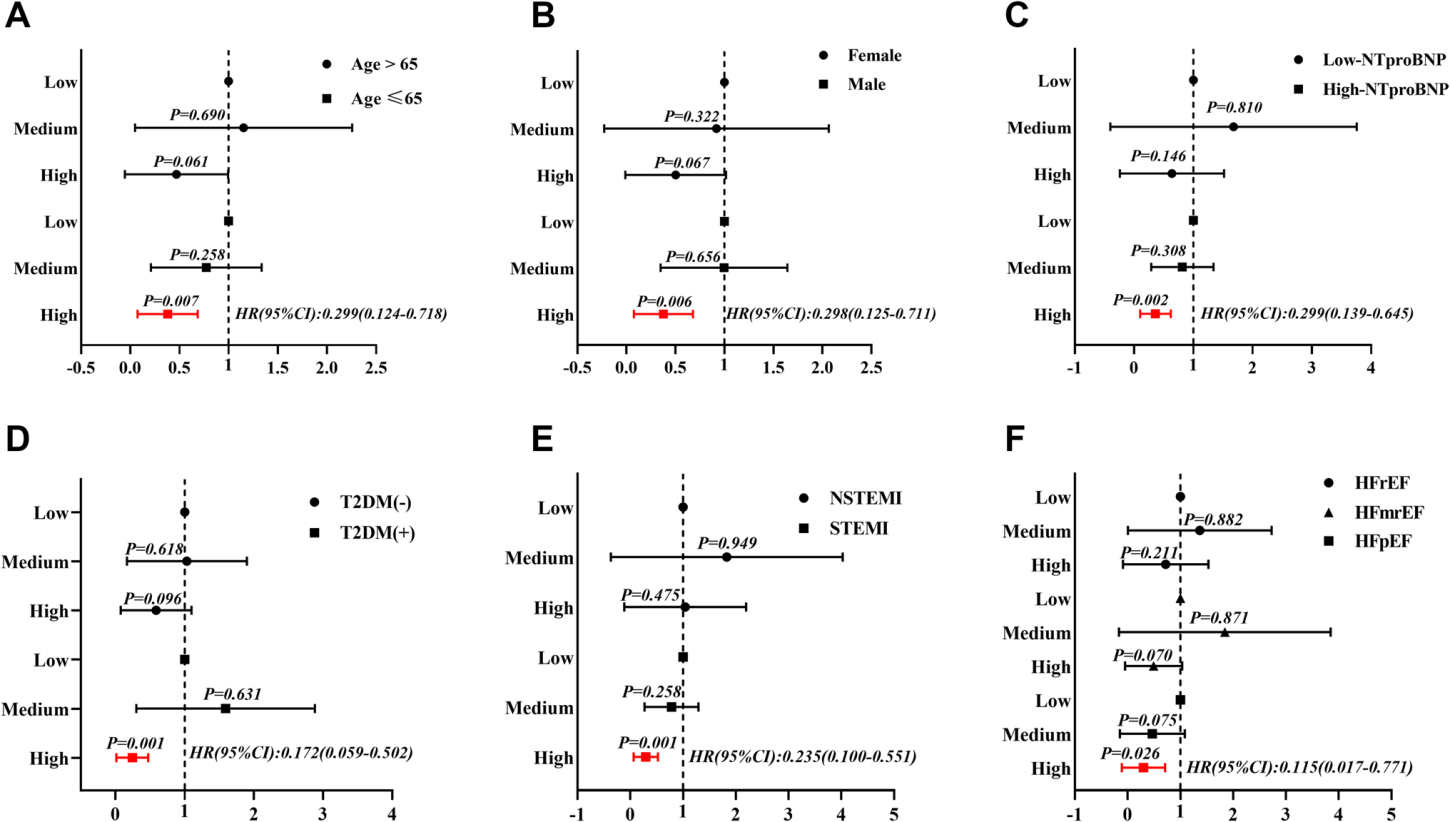
Classification of subgroups: (**A**) 407 patients by age ≤ 65 years vs. age > 65 years; (**B**) Male vs. Female; (**C**) High NT-proBNP vs. Low NT-proBNP (NT-proBNP ≥ 300 pg/mL was defined as High, otherwise as Low); (**D**) T2DM vs. non-T2DM; (**E**) STEMI vs. NSTEMI, and (**F**) 267 patients with HF during hospitalization by HFrEF vs. HFmrEF vs. HFpEF

Subgroup multivariable COX regression analysis (Fig. 4 A ~ F) showed that the predictive value of 5-MTP for 1-year MACE was more significant in patients ≤ 65 years (HR = 0.299, 95%Cl 0.124–0.718, *p* = 0.007), males (HR = 0.298, 95%Cl 0.125–0.711, *p* = 0.006) and those with high NT-proBNP (HR = 0.299, 95%Cl 0.139–0.645, *p* = 0.002), T2DM (HR = 0.172, 95%Cl 0.059–0.502, *p* = 0.001), STEMI (HR = 0.235, 95%Cl 0.100–0.551, *p* = 0.001), and baseline HFpEF (HR = 0.115, 95%Cl 0.017–0.771, *p* = 0.026) characteristics.

## Discussion

To the best of our knowledge, this is the first study to show the prognostic value of 5-MTP in patients with AMI undergoing PCI. We found that (1) Patients with AMI had a negative correlation between plasma 5-MTP and NT-proBNP as well as a positive correlation with LVEF. (2) After 1-year follow-up, a significant downtrend in the incidence of MACE and HF was observed as the 5-MTP level increased (All Log-rank *p* < 0.05); the ROC curve analysis revealed that 5-MTP had superior AUC for predicting MACE or HF, and after adjusting for several traditional risk factors, high 5-MTP concentration could reduce the 1-year incidence of MACE (HR = 0.33, 95%Cl 0.17–0.64, *p* = 0.001), and HF (HR = 0.28, 95%Cl 0.13–0.62, *p* = 0.002) in comparison with low 5-MTP concentrations, and (3) Subgroup analysis showed that the predictive value of 5-MTP for 1-year MACE was more significant in patients aged ≤ 65 years, males, and those with higher baseline NT-proBNP, T2DM, STEMI, and baseline HFpEF characteristics. This will provide valuable information for the early risk stratification post-MI.

Post-MI outcomes depend significantly on complex interactions between several cellular mechanisms, including inflammatory, oxidative, and apoptotic pathways. Following MI, adverse remodeling may result in worsened outcomes and higher patient mortality due to reduced LVEF, chamber dilatation, and increased fibrosis. Inflammation and fibrosis are the main repair mechanisms of tissue injury. However, uncontrolled extracellular matrix (ECM) deposition in the interstitial space between the injured and normal tissues may lead to structural remodeling and functional defects [21]. Following an acute coronary artery occlusion, ischemia, and ischemia–reperfusion injury induce cardiomyocyte necrosis and apoptosis. The damaged cells then release chemokines such as CXCL2 and CCL2, which can draw monocytes to the damaged site and differentiate them into macrophages, thereby infiltrating the injured and adjacent normal tissues and causing inflammation [22]. Through their interactions, they stimulate fibroblasts to differentiate into myofibroblasts, which release collagen and ECM proteins to cause myocardial fibrosis (MF). Thus, macrophage-mediated inflammatory tissue injury and MF are the primary pathophysiological processes that lead to LVR and HF after MI [9, 23].

In vivo and in vitro studies have shown that 5-MTP inhibits macrophage activation by hampering the activation of NF-KB mediated by p38-MAPK and blocking the release of pro-inflammatory cytokines, chemokines, and COX-2 expressions by macrophages [24, 25]. Therefore, 5-MTP is considered a novel weapon against inflammation [26] as it helps in controlling fibroblast differentiation and pathological fibrosis in important organs and might prove very useful in this line of treatment.

Animal experiments have found that 5-MTP can maintain vascular barrier function and the endothelial system’s integrity as well as inhibit inflammatory cell adhesion and vascular hyperpermeability. A study on the mouse carotid artery injury model suggested that 5-MTP inhibits vascular injury-induced vascular endothelial and smooth muscle cell proliferation [27]. In the mouse model of AMI caused by the anterior descending branch ligation, it was found that an intraperitoneal 5-MTP injection could reduce myocardial oxidative stress, ischemia–reperfusion injury, infarct area, and fibrosis as well as restore myocardial function in comparison with the blank and saline control groups, respectively [13]. Shuai et al. [28] found that 5-MTP administration decreases the vulnerability of ibrutinib-related atrial fibrillation, caused by ameliorated maladaptive left atrial remodeling and calcium handling protein dysregulation.

Additionally, Lin et al. [29] showed that children with lupus nephritis who had greater baseline 5-MTP levels were more likely to achieve complete remission after immunomodulatory therapy. Ko et al. [30]. found that 5-MTP can be used as a biomarker to reflect the long-term prognosis of hepatocellular carcinoma patients. Another study by Hong et al. [31] suggested that 5-MTP treatment can reduce neuroinflammation in spinal cord trauma caused by microglia.

To date, the only clinical cardiovascular study carried out by Lin et al. [14] revealed that the 5-MTP level on day 3 after AMI correlated with the NT-proBNP level 1 year later, but without clinical events as the observation endpoint. After using MACE as our endpoint, we found that AMI patients with lower 5-MTP baseline concentration were more likely to have long-term HF readmission adverse events, while no significant differences were observed in other MACE components like all-cause death, non-fatal recurrent MI, and TLR. Hence, this suggested that 5-MTP might act as a protective factor for HF and can become a new target for the prediction as well as treatment of HF after MI.

Subgroup analysis showed that the predictive value of 5-MTP for 1-year MACE was more significant in patients aged ≤ 65 years, males, and those with higher baseline NT-proBNP, T2DM, STEMI, and baseline HFpEF characteristics. This may suggest that 5-MTP related inflammatory mediators and antifibrotic pathways are significantly involved in LVR after MI in this subgroup of patients and young patients(≤ 65 years) with AMI and baseline HFpEF characteristics might benefit from early suppression of inflammation and anti-fibrosis.

Zasada et al. [32] found that younger patients with AMI have more non-atherosclerotic mechanisms and fewer traditional risk factors such as hypertension, diabetes, and CKD than older patients with AMI. A study by Jortveit et al. [33] suggested that approximately one in ten young patients with AMI died or experienced a new cardiovascular event during follow-up. Thus, increased efforts should be undertaken to improve risk factor control in young patients with AMI. Hence, our study may provide new perspectives for risk stratification and prognosis improvement in young patients with AMI.

Diabetic patients exhibit an inflammatory response, widespread and multi-branch lesions, and a worse prognosis. Compared to NSTEMI patients, STEMI patients mostly have transmural MI with a more pronounced LVR process. Thus, we suggest that 5-MTP might exhibit protective effects on diabetic and STEMI patients, who are more likely to benefit from early inflammation inhibition and anti-fibrosis therapy.

Since HFpEF may occur in AMI patients, particular attention should be given to HFpEF in conjunction with lower 5-MTP levels, our study may suggest beneficial for the early anti-inflammatory and anti-fibrosis treatment of this subgroup, aligning with the previous findings from animal experiments [13],which found that early 5-MTP treatment constricted the infarct area while an additional 5-MTP dose administered twice weekly provided no extra benefits.

## Limitations

Firstly, this was a single-center cohort study with a relatively small sample size. The 5-MTP concentration trend was not continuously monitored and the impact of the variations in 5-MTP concentration on the long-term prognosis of AMI was not assessed adequately. Secondly, because biomarkers like NT-proBNP and echocardiography were not detected at the end of follow-up, the effect of 5-MTP on LVR after MI was not assessed. Thirdly, HFrEF, HFmrEF, and HFpEF could not be used to categorize HF throughout the follow-up.

Further studies on the predictive value of 5-MTP for long-term HF after PCI in patients with MI need to be conducted with a larger sample size and multiple centers to investigate the relationship between 5-MTP and LVR, inflammation, gut flora, and thrombus. The relevant mechanisms, including inflammation, fibrosis, and oxidative stress, should be thoroughly investigated in cellular and animal experiments. Explore new methodology for plasma 5-MTP detection. The molecular mechanisms of 5-MTP and the relevant pathways can also be explored by mass spectrometry tryptophan omics and bioinformatics analysis to understand to provide the basis for 5-MTP to be the target of prevention and treatment of HF after MI.

## Conclusion

As a protective factor, plasma 5-MTP is a potential independent early biomarker for 1-year MACE and HF events in patients with AMI after discharge, especially for younger patients and those with T2DM, STEMI, and baseline HFpEF characteristics.

## Supplementary Information

Below is the link to the electronic supplementary material.

Supplementary file1 (DOCX 16 KB)

## Data Availability

The experimental data and the simulation results that support the findings of this study are available in Science Date Bank with the identifier: https://download.scidb.cn/download?fileId=252d8787912ebeff72cd9bcda9ed7582&dataSet Type = undefined&username = hkuian@126.com&fileName = database.sav.

## Declarations

We certify that all authors have seen and approved the manuscript being submitted, have contributed significantly to the work, attest to the validity and legitimacy of the data and its interpretation, and agree to its submission to the Journal of *Cardiovascular Translational Research*. We attest that the article is the Authors' original work, has not received prior publication and is not under consideration for publication elsewhere. On behalf of all Co-Authors, the corresponding Author bear full responsibility for the submission.
