# Supplementary material for: Predictive Value of 5-Methoxytryptophan on Long-Term Clinical Outcome after PCI in Patients with Acute Myocardial Infarction-a Prospective Cohort Study
Source: J Cardiovasc Transl Res. 2024 Apr 29;17(5):1036–47. doi: 10.1007/s12265-024-10518-6 (PMC11519123; doi:10.1007/s12265-024-10518-6)
Supplement: Supplementary file 1 — Supplementary file1 (DOCX 16 KB) [file 12265_2024_10518_MOESM1_ESM.docx]

supplementary table 1.

Multivariable COX regression analysis of the factors influencing HF 1 year after AMI

| Variables | groups | *B* | *SE* | *Wald* | *P* | HR | 95%CI |
| --- | --- | --- | --- | --- | --- | --- | --- |
| Gender | Female | 0.336 | 0.347 | 0.936 | 0.333 | 1.399 | 0.709-2.762 |
|  | Male* |  |  |  |  |  |  |
| Age |  | 0.004 | 0.012 | 0.102 | 0.749 | 1.004 | 0.98-1.028 |
| Hypertension | Yes | 0.309 | 0.319 | 0.942 | 0.332 | 1.362 | 0.73-2.543 |
|  | No* |  |  |  |  |  |  |
| Diabetes | Yes | 1.229 | 0.324 | 14.409 | ＜0.001 | 3.419 | 1.812-6.45 |
|  | No* |  |  |  |  |  |  |
| Previous MI | Yes | -0.008 | 0.37 | 0 | 0.984 | 0.992 | 0.481-2.05 |
|  | No* |  |  |  |  |  |  |
| NT-proBNP | High | 2.09 | 0.493 | 17.959 | ＜0.001 | 8.085 | 3.075-21.256 |
|  | Low* |  |  |  |  |  |  |
| Cr |  | 0.009 | 0.005 | 3.731 | 0.053 | 1.009 | 1-1.018 |
| hsCRP |  | 0.017 | 0.008 | 5.064 | 0.024 | 1.017 | 1.002-1.032 |
| LVEF |  | -0.102 | 0.021 | 23.722 | ＜0.001 | 0.903 | 0.867-0.941 |
| Triple-vessel disease | Yes | 0.448 | 0.408 | 1.201 | 0.273 | 1.565 | 0.703-3.484 |
|  | No* |  |  |  |  |  |  |
| Left main disease | Yes | 0.254 | 0.531 | 0.229 | 0.632 | 1.29 | 0.455-3.655 |
|  | No* |  |  |  |  |  |  |
| 5-MTP | Low* |  |  |  |  |  |  |
|  | Medium | -0.263 | 0.343 | 0.586 | 0.444 | 0.769 | 0.393-1.506 |
|  | High | -1.277 | 0.407 | 9.843 | 0.002 | 0.279 | 0.126-0.619 |

*Denotes reference group; NT-proBNP≥300 pg/mL was defined as High, otherwise as Low; MI, myocardial infarction; NT-proBNP, N-Terminal pro-brain natriuretic peptide; Cr, [creatinine](javascript:;); hsCRP, high sensitivity C-reactive protein; LVEF, left ventricular ejection fraction.
